# A New Model of Biodosimetry to Integrate Low and High Doses

**DOI:** 10.1371/journal.pone.0114137

**Published:** 2014-12-02

**Authors:** Mònica Pujol, Joan-Francesc Barquinero, Pedro Puig, Roser Puig, María Rosa Caballín, Leonardo Barrios

**Affiliations:** 1 Unitat d’Antropologia Biològica, Departament de Biologia Animal, Biologia Vegetal i Ecologia, Universitat Autònoma de Barcelona, Bellaterra, Spain; 2 Departament de Matemàtiques, Universitat Autònoma de Barcelona, Bellaterra, Spain; 3 Unitat de Biologia Cellular, Departament de Biologia Cel·lular, Fisiologia i Immunologia, Universitat Autònoma de Barcelona, Bellaterra, Spain; Institute for Health & the Environment, United States of America

## Abstract

Biological dosimetry, that is the estimation of the dose of an exposure to ionizing radiation by a biological parameter, is a very important tool in cases of radiation accidents. The score of dicentric chromosomes, considered to be the most accurate method for biological dosimetry, for low LET radiation and up to 5 Gy, fits very well to a linear-quadratic model of dose-effect curve assuming the Poisson distribution. The accuracy of this estimation raises difficulties for doses over 5 Gy, the highest dose of the majority of dose-effect curves used in biological dosimetry. At doses over 5 Gy most cells show difficulties in reaching mitosis and cannot be used to score dicentric chromosomes. In the present study with the treatment of lymphocyte cultures with caffeine and the standardization of the culture time, metaphases for doses up to 25 Gy have been analyzed. Here we present a new model for biological dosimetry, which includes a Gompertz-type function as the dose response, and also takes into account the underdispersion of aberration-among-cell distribution. The new model allows the estimation of doses of exposures to ionizing radiation of up to 25 Gy. Moreover, the model is more effective in estimating whole and partial body exposures than the classical method based on linear and linear-quadratic functions, suggesting their effectiveness and great potential to be used after high dose exposures of radiation.

## Introduction

In cases of accidental exposures to ionizing radiation (IR), it is very important to estimate the dose received to guide medical decisions. When physical measurements are not available or it is suspected that dosimeters have not been used correctly, biodosimetric methods become necessary to obtain a precise knowledge of the received dose for a clear evaluation of the case. Within the biodosimetric methods, the score of dicentric chromosomes in metaphases of peripheral blood lymphocytes, cultured around 48 h, is widely considered as the “gold standard” method that accurately estimates doses in cases of acute and recent exposures [1]. This is due to the very low background frequency of dicentrics and to the strong relationship between the frequency of dicentrics and the dose. Currently, the majority of dose-effect curves for dicentric chromosomes include doses from 0 to 5 Gy. For this dose range and for low LET radiation types, such as X and gamma rays, the dose-effect relationship fits well to a linear-quadratic model. Additionally after whole body exposure from 0 to 5 Gy the distribution of dicentrics among cells agrees with the Poisson distribution, allowing the detection of partial body exposures when deviations of the Poisson are detected [2], [3], [4].

Some accidents have demonstrated the need to evaluate exposures to high doses and if they are whole or partial body exposures [5], [6], [7]. Nevertheless, the dicentric based biodosimetry becomes less suitable for doses of IR higher than 5 Gy, because the number of cells able to reach metaphase decreases dramatically when the dose increases. After a high dose exposure heavily damaged cells, which usually bear incomplete chromosome aberrations, show a delay or even the impossibility of progressing through the G2/M cell cycle checkpoint to reach mitosis [1], [8]. To obtain enough metaphases at doses over 5 Gy, some authors have increased the culture time allowing delayed cells to reach mitosis, but in some cases the adjustment to linear-quadratic dose-effect curves showed clear deviations from the aberration frequencies observed [9], [10]. In other studies, a better fit to the linear-quadratic model has been obtained but with negative α and/or β coefficients [6], [11]. To achieve a better adjustment for very high doses a multiparametric model based on modifications on the linear-quadratic model was proposed by Sasaki [9].

A preliminary study using caffeine to evaluate its suitability for the analysis of dicentric chromosomes after high doses of IR showed that the mitotic index in caffeine treated cultures was good enough for dose assessment [12]. In fact, caffeine treatment abrogates the G2/M checkpoint, and increases the number of damaged cells that progress until metaphase [13], [14], [15]. In the present study we have standardized the optimal culture time and caffeine treatment for the analysis of dicentrics irradiating at doses from 0 to 25 Gy. We propose a new integrated model for dose-effect calibration, based on a specific weighted Poisson distribution of dicentrics and adjusting the observed values to a Gompertz-type function. Finally, and to test the model, simulated whole and partial body irradiations have been assessed.

## Materials and Methods

### Irradiation conditions

Two donors, a 24-year-old female and a 53-year-old male with no history of exposure to clastogenic agents, signed an informed consent following the ethical guidelines for good practice of the *Universitat Autònoma de Barcelona* before blood extraction. Moreover, the Ethics Committee on Animal and Human Research of the *Universitat Autònoma de Barcelona* informed that the present research project “does not require an approval by the ethics committee”. Irradiations for three different purposes were performed: One, to standardize the caffeine treatment; second, to elaborate the dose-effect curve; and a third, to simulate whole and partial body irradiations. In all cases blood samples were obtained by venipuncture and collected in heparinized tubes less than one hour before irradiation. To standardize the treatment with caffeine, peripheral blood samples from the 24-year-old female were irradiated at 10 Gy (dose rate of 5.25 Gy.min^-1^) using a 137Cs source (IBL437C, CIS Biointernational, GIF Yvette, France) located at the *Unitat Tècnica de Protecció Radiològica* of the Universitat *Autònoma de Barcelona*. During irradiations, IAEA recommendations were followed (IAEA 2011). For the dose-effect curve elaboration, blood samples from the same donor were irradiated, in the same conditions described above, at 0, 0.1, 0.5, 1, 3, 5, 7, 10, 15, 20 and 25 Gy. To simulate whole and partial body irradiations peripheral blood samples from the 53-year-old male were obtained and irradiated at 2, 6, 12 and 17 Gy in the same conditions. To simulate partial body exposures, irradiated blood at 6 and 12 Gy was mixed with non-irradiated to obtain fractions of 30% and 70% of irradiated blood.

### Culture conditions and harvesting

Lymphocytes were cultured in Roswell Park Memorial Institute (RPMI) 1640 medium (GIBCO, Life Technologies, Madrid, Spain) supplemented with 15% fetal calf serum (GIBCO), 1% of L-glutamine 200 mM (GIBCO), antibiotics (100 IU·mL^−1^ penicillin, 100 µg·mL^-1^ streptomycin) (GIBCO), 1∶1000 of heparin (ROVI, ROVI S.A., Madrid, Spain), and 4% of phytohemagglutinin (PHA) (GIBCO). For all cultures, 0.1 µg·mL^−1^ Colcemid (GIBCO) was added 24 h after the culture set up to analyze only first division cells. Caffeine (Sigma-Aldrich Química, Madrid, Spain) was added at 46 h of incubation at a final concentration 0.3 µg·mL^−1^. To standardize the optimal treatment with caffeine, samples irradiated at 10 Gy were cultured up to 48, 51, 54, 57, 60, 63 and 72 hours. For the elaboration of the dose-effect curve and the simulations of whole and partial body exposures the cultures were harvested at optimal culture time of 57 h. All cultures were harvested using the standard treatment with hypotonic and Carnoy’s fixative. Slides were stained with Leishman stain (Leishman eosin methylene blue solution modified, Merck, Madrid, Spain).

### Microscope analysis

Chromosome analyses were carried out exclusively in metaphases containing 46 centromeres. In the standardization of the caffeine treatment, 150 metaphases were analyzed at each culture time. For the elaboration of the dose-effect curve, 2000 metaphases were scored at doses of 0, 0.1 and 0.5 Gy, 1000 at 1 Gy, 500 at 3 Gy, 150 at 5, 7 and 10 Gy and 100 at 15, 20 and 25 Gy. For the whole and partial body exposure simulations, a minimum of 100 dicentrics or 100 cells were scored in blind samples. Multicentric chromosomes (di-, tri-, tetra-…) were only recorded when the corresponding number of acentric fragments was present. Polycentrics were converted into the equivalent number of dicentrics as (n –1), where n is a number of centromeres. All cells with doubts or with dicentrics were analyzed by two scorers. In the standardization of the caffeine treatment the mitotic index (MI) was determined as the ratio of the number of metaphases in 500 stimulated nuclei [1].

### Statistics

To evaluate if the distribution of dicentrics among cells followed a Poisson, the u-test was used [16]. U values out of the interval ±1.96 indicate that the dicentric cell distribution does not follow a Poisson with a 5% level of significance. As will be described in the results, our data presented a significant underdispersion. Therefore, a new count probability function has been considered to model our underdispersed count data, having the form,
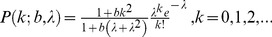
(1)


This is a specific weighted Poisson distribution [17] with a weight equal to 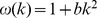, representing the sighting mechanism. This increasing function of k gives more weight to the large values than to the small ones. This two-parameter distribution is very similar to the PL_2_ distribution described in [18] but, for our data set, the performance of (1) is better.

The domain of the parameters is 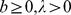 and for 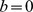 this is just the Poisson probability function. Direct calculations show that the expectation (population mean) and variance have the form,
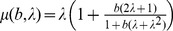
(2)




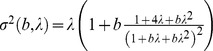



Changing the values of the parameters 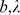, the dispersion index 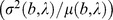 can take values slightly greater than 1 (around to 1.1) and values lower than 1. Therefore, the probability distribution described in (1) is useful to model count data presenting underdispersion, such as that observed in the empirical dicentrics distributions for the establishment of the dose-effect curve shown in results section. It is important to remark that this is an empirical solution, not an explanation of why the underdispersion occurs. To construct the dose-effect curve, we have considered parameter 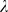 to be dependent of the dose *d*, following a Gompertz curve of the form,
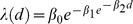



This is a flexible curve that allows sigmoid patterns to be fitted. Moreover, parameter *b* has also been considered depending on the dose in a simple linear form 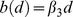. Replacing 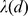 and 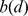 in the expression of the population mean (2), we obtain the profile of the curve which describes the frequency of dicentrics as a function of the dose, that will be denoted as 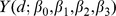. This is a sigmoid curve with a profile very similar to that of the Gompertz curve, which from now on will be indicated as GT (from Gompertz-type). Therefore, in order to estimate the dose-effect GT-curve, four parameters have to be estimated. This has been done by means of the maximum likelihood method, with a program made in R using the procedure nlm (supporting information S1). The parameter estimates will be denoted as 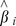, and the estimated dose-effect GT-curve as 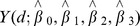 or in a short form 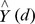. The 95% confidence limits of the curve have the form 
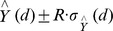
, where 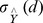 is the estimated standard error of a prediction for a dose equal to d, specifically calculated for our model using the delta-method. The constant *R* is the square root of the 0.95 quantile of a chi-square distribution with four degrees of freedom (the number of parameters). Given a blood sample with a frequency of dicentrics *Y_0_* (this is the observed yield), a point estimation of the received dose *d_0_* is obtained solving numerically the equation 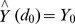. The confidence limits of the estimated dose (*d_L_,d_U_*) are calculated using a version of the inverse regression Merkle’s approach proposed into IAEA manual [1], which consists of solving numerically the equations 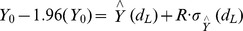 and 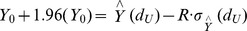, where 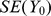 is the standard error of the observed yield.

Partial body irradiation produces distributions of dicentrics that are zero-inflated and this also increases the dispersion index of the distribution. However, because our distributions are underdispersed, for high fraction volumes of irradiated blood this increment could be insufficient to generate overdispersion. Consequently, the u-test has to be used with caution. To estimate the received dose in partial body irradiation scenarios, the empirical distribution of dicentrics of the blood sample has been fitted by maximum likelihood to a zero-truncated version of the probability function shown in (1), that is, 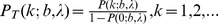, obtaining 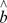 and 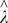. The estimated yield is calculated as 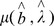, using expression (2), and its standard error is obtained using the delta-method. The estimated dose and its confidence limits are calculated using again the version of the Merkle’s approach described above, but taking the estimated yield 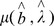 and its standard error.

In order to compare our method with the classical ones, the linear and linear-quadratic dose-effect models were also fitted by maximum likelihood, using the usual Poisson distribution assumption for dose assessment. After whole body exposure simulations the inverse regression Merkle’s approach was used [1] and for partial body exposure simulations the Dolphin method [1] was used to calculate the expected yield in the irradiated fraction. In both cases 95% confidence interval was calculated using the Merkle’s approach [1] as in GT.

## Results

### Standardization of caffeine treatment

**Figure 1:**
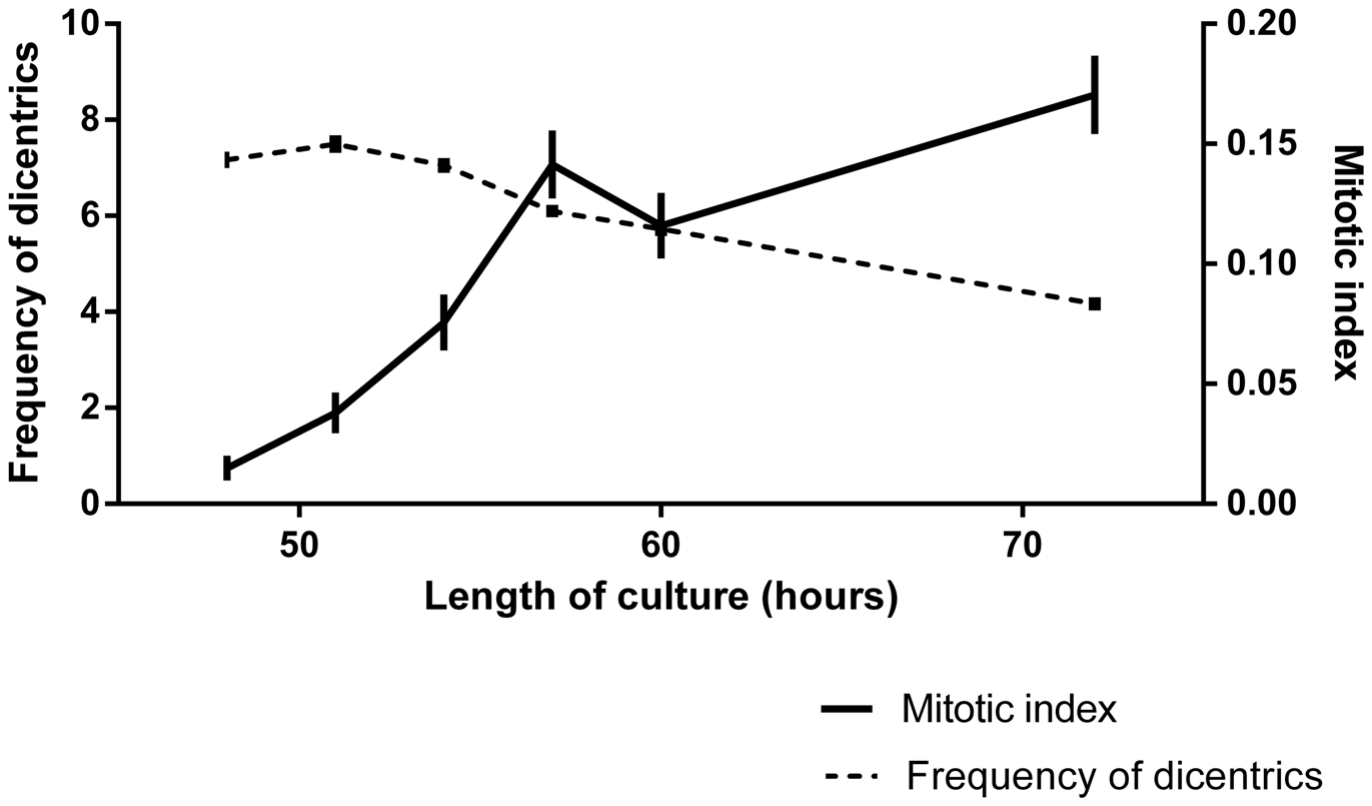
Frequency of dicentrics and mitotic index after irradiation at 10 Gy and at different culture times with caffeine treatment. Error bars indicate the SEM.

As can be seen in Figure 1 and Table 1, after 10 Gy irradiation and for 48, 51 and 54 hours of culture, the frequency of dicentrics remained constant around 7 dicentrics per cell. Then, the frequency started to decrease, 6 at 57 h of culture and 4 at 72 h. Inversely the MI increased during the first four culture times, from 2 (48 h) to 14 (57 h) metaphases per hundred cells, remaining relatively constant at longer culture times (14 and 17 metaphases per hundred cells at 57 and 72 hours of culture). As can also be seen, at all culture times the distribution of cells with dicentrics showed a significant underdispersion (U value lower than −1.96).

**Table 1:** Culture time, cells analyzed and dicentrics distribution among cells for the standardization of the caffeine treatment after irradiation at 10 Gy.

			Dicentrics distribution among cells																				
Culture time	Cells	0	1	2	3	4	5	6	7	8	9	10	11	12	13	14	15	Total Dic	Y	Var	SE	DI	U
48h	150	0	0	0	3	10	20	28	32	17	16	18	3	1	0	1	1	1075	7.167	4.502	0.173	0.63	−3.21
51h	150	0	0	0	1	8	23	21	30	22	17	10	10	6	1	1	0	1124	7.493	4.963	0.182	0.66	−2.92
54h	150	0	0	0	0	13	21	31	24	25	20	9	7	0	0	0	0	1058	7.053	3.554	0.154	0.50	−4.28
57h	150	0	0	0	3	18	40	35	25	16	9	4	0	0	0	0	0	915	6.100	2.493	0.129	0.41	−5.11
60h	150	0	0	3	18	27	16	34	26	11	12	3	0	0	0	0	0	860	5.733	3.687	0.157	0.64	−3.08
72h	150	0	3	17	40	37	23	16	9	2	2	0	0	1	0	0	0	625	4.167	2.985	0.141	0.72	−2.45

In all cultures Caffeine was added 46 h after the set up.

Total dic = total number of dicentrics; Y = frequency of dicentrics; Var = variance; SE = standard error; DI = dispersion index (variance/mean); U = values of the u-test.

### Establishment of the dose-effect curve

**Figure 2:**
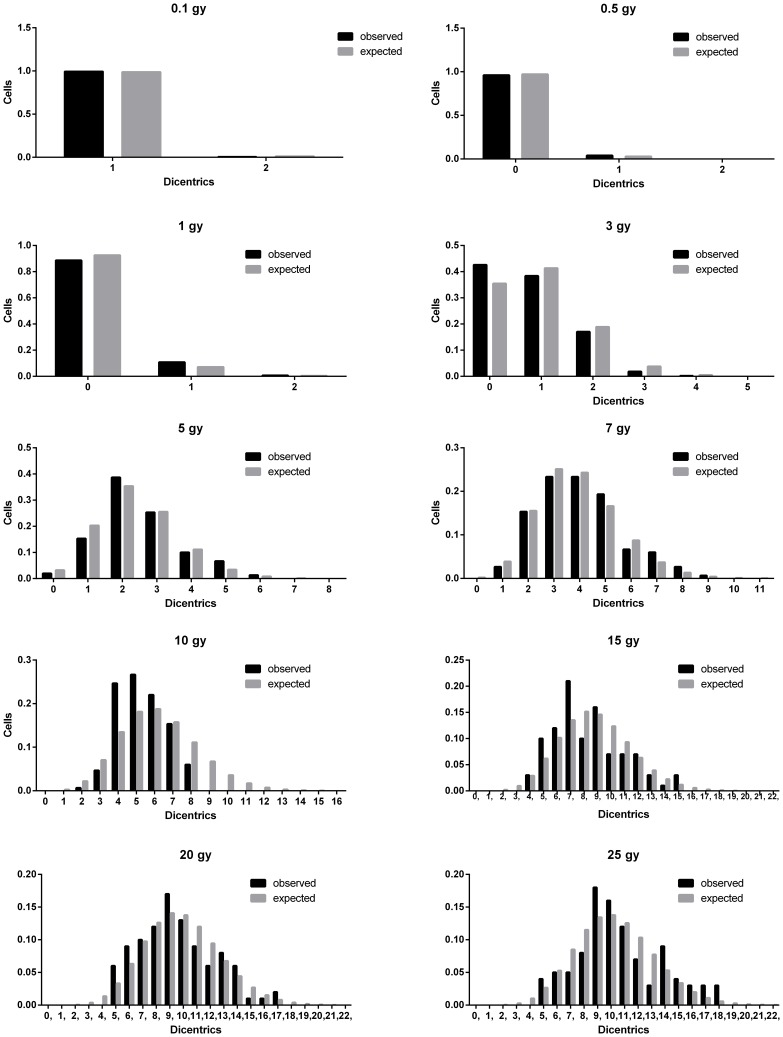
Observed distribution of dicentrics among cells. The expected cell distribution was calculated using the GT model and the weighted Poisson.

The results used for the elaboration of the dose-effect curve are shown in Table 2. As expected a clear increase in the frequency of dicentrics was observed as the dose increased, being more accused at the low doses. For example the frequency of dicentrics per cell increased by more than twice between 5 and 10 Gy, from 2.5 to 6.1, whereas between 10 and 20 Gy the frequency only increased from 6.1 to 9.6 dicentrics per cell. The compliance of dicentrics cell distribution with Poisson distribution was not rejected for six of the 10 doses evaluated. However, in all cases U values were negative and for 3, 5, 7 and 10 Gy U values indicated a significant underdispersion. The expected cell distribution of dicentrics, assuming a Poisson and considering the sample mean observed, is shown in brackets in Table 2. As can be seen, a clear tendency to detect fewer cells without or with fewer dicentrics than expected under the Poisson assumption was observed as the dose increased. At 5 Gy the number of cells without dicentrics was lower than expected. At 7 Gy no cells without dicentrics were observed and the number of cells with 1 dicentric was lower than expected. At 10 Gy, no cells without dicentrics were observed and fewer cells than expected had 3 or 4 dicentrics. At 20 and 25 Gy no cells with 3, 4 dicentrics were observed. This behavior has been conveniently modeled using the weighted Poisson distribution described in (1) (Figure 2). When the higher classes are evaluated it seems that the number of cells with many dicentrics was also underrepresented. This was clear at 5, 7 and 10 Gy. However at 20 and 25 Gy this effect was not clearly observed.

**Table 2:** Cells analyzed and dicentrics distribution among cells for the dose-effect curve.

		Dicentrics distribution among cells																						
Dose (Gy)	Cells	0	1	2	3	4	5	6	7	8	9	10	11	12	13	14	≥15#	Total Dic	Y	Var	SE	DI	U	
0	2000	1999	1	0	0	0	0	0	0	0	0	0	0	0	0	0	0	1	0.001	0.001	0.001	1.00	–	
		(1998)	(2)	0	0	0	0	0	0	0	0	0	0	0	0	0	0							
0.1	2000	1989	11	0	0	0	0	0	0	0	0	0	0	0	0	0	0	11	0.006	0.005	0.002	0.99	−0.17	
		(1988)	(12)	0	0	0	0	0	0	0	0	0	0	0	0	0	0							
0.5	2000	1922	78	0	0	0	0	0	0	0	0	0	0	0	0	0	0	78	0.039	0.037	0.004	0.96	−1.23	
		(1924)	(75)	(1)	0	0	0	0	0	0	0	0	0	0	0	0	0							
1	1000	886	108	6	0	0	0	0	0	0	0	0	0	0	0	0	0	120	0.120	0.118	0.011	0.98	−0.43	
		(887)	(106)	(6)	0	0	0	0	0	0	0	0	0	0	0	0	0							
3	500	213	192	85	9	1	0	0	0	0	0	0	0	0	0	0	0	393	0.786	0.641	0.036	0.82	−2.91	
		(228)	(179)	(70)	(18)	(4)	(1)	0	0	0	0	0	0	0	0	0	0							
5	150	3	23	58	38	15	10	2	1	0	0	0	0	0	0	0	0	382	2.547	1.578	0.103	0.62	−3.29	
		(12)	(30)	(38)	(32)	(21)	(10)	(4)	(2)	(1)	0	0	0	0	0	0	0							
7	150	0	4	23	35	35	29	10	9	4	1	0	0	0	0	0	0	604	4.027	2.697	0.134	0.67	−2.85	
		(3)	(11)	(22)	(29)	(29)	(24)	(16)	(9)	(5)	(2)	(1)	0	0	0	0	0							
10	150	0	0	0	3	18	40	35	25	16	9	4	0	0	0	0	0	915	6.100	2.493	0.129	0.41	−5.11	
		(1)	(4)	(11)	(19)	(25)	(26)	(23)	(17)	(12)	(7)	(4)	(2)	(1)	0	0	0							
15	100	0	0	0	0	3	10	12	21	10	16	7	7	7	3	1	3	834	8.340	6.792	0.261	0.81	−1.31	
		0	0	(1)	(2)	(5)	(8)	(11)	(13)	(14)	(13)	(11)	(8)	(6)	(4)	(2)	(2)							
20	100	0	0	0	0	0	6	9	10	12	17	13	9	6	8	6	4	957	9.570	7.985	0.283	0.83	−1.17	
		0	0	0	(1)	(2)	(5)	(7)	(10)	(12)	(13)	(12)	(11)	(9)	(6)	(4)	(6)							
25	100	0	0	0	0	0	4	5	5	8	18	16	12	7	3	9	13	1065	10.650	10.008	0.316	0.94	−0.42	
		0	0	0	(1)	(3)	(5)	(7)	(10)	(12)	(12)	(12)	(11)	(9)	(7)	(5)	(8)							

In brackets are shown the expected dicentrics distribution assuming a Poisson.

Gy = Gray; Total dic = total number of dicentrics; Y = frequency of dicentrics; Var = variance; SE = standard error; DI = dispersion index (variance/mean); U = values of the u-test. # = Extended data: At 15 Gy, three cells with 15 dic were observed and one cell with 15 and 16 dic were expected; At 20 Gy, we observed one cell with 15 dic, one with 16 and two with 17. At this dose three cells with 15, two with 16 and one with 17 dic were expected. At 25 Gy we observed four cells with 15 dic, three with 16, three with 17, and three with 18. At this dose we expected three cells with 15 dic, two with 16 and one with 17, 18 and 19 respectively.

**Figure 3:**
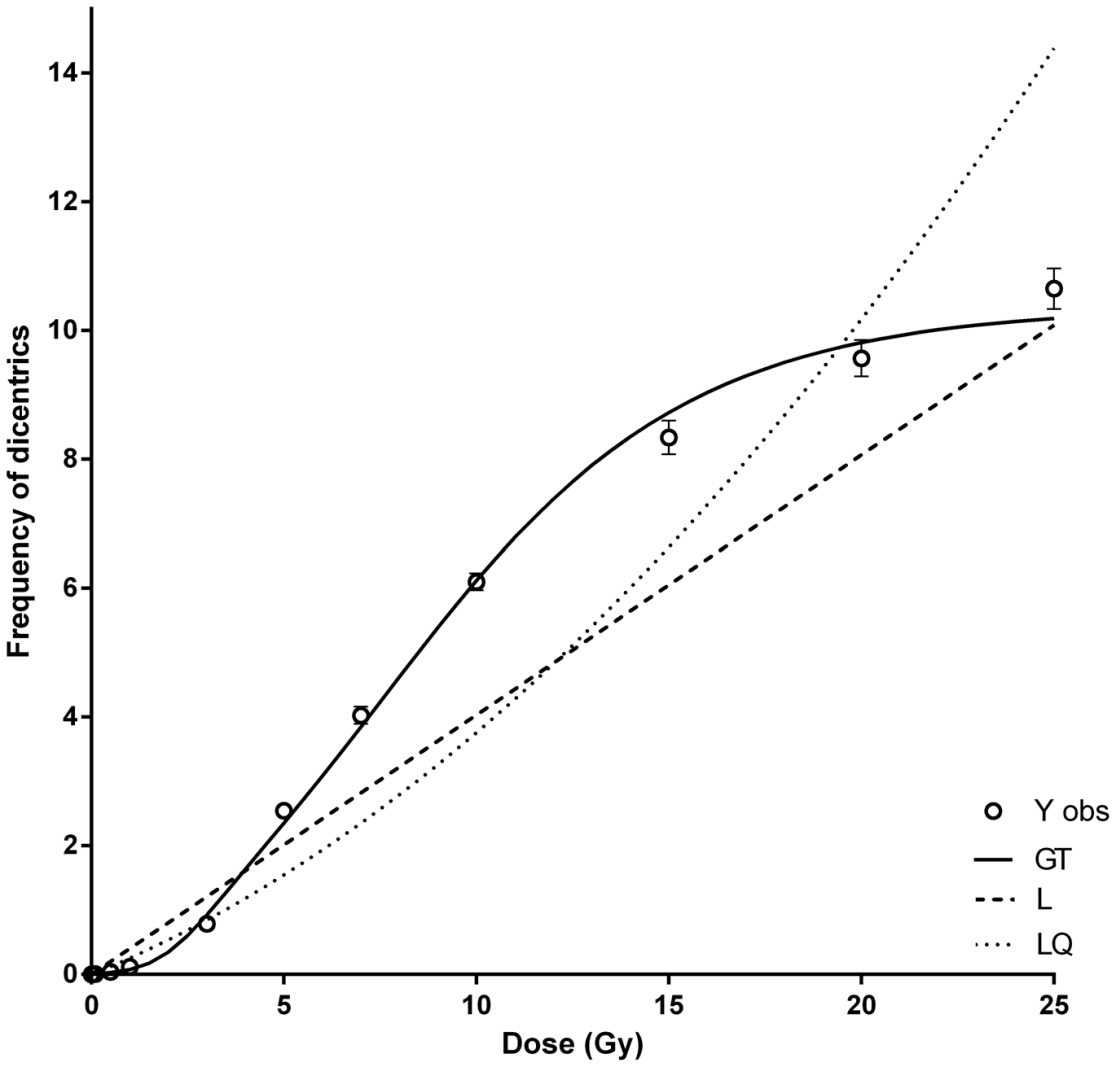
Frequencies of dicentrics (Y obs) and their fit to the linear (L), linear quadratic (LQ) and Gompertz-type (GT) models. Error bars indicate the SEM.

In Table 3 are shown the coefficients of the fitting to the linear, linear quadratic and GT models. Details of the “dose_response_curve.txt” program output for the GT model fitting can be seen in Figures S1 and S2 in File S1. In the case of the linear and linear-quadratic models, the basal frequency coefficient (C) was negative and for this reason the fitting was carried out exclusively for the linear and linear-quadratic coefficients. Additionally and to explain the underdispersion and the saturation in the frequency of dicentrics observed at the highest doses, the frequencies of dicentrics were adjusted to a GT model, based on the weighted Poisson distribution defined in (1), where fewer differences between the observed dicentric distribution and the expected one for the model were achieved for cells with no or few dicentrics (Figure 2). The fitting of the observed frequencies to the three models can be seen in Figure 3. The χ2–statistic was used to compare the different adjustments (Table 3), indicating a better adjustment for the GT model. Details of the “wholebody_dose.txt” and “partialbody_dose.txt” programs outputs for whole and partial body dose estimations respectively, can be seen in Figures S3 and S4 in File S1.

**Table 3:** Dose-response coefficients obtained for the different adjustments to the models and their goodness-of-fit χ^2^ statistics.

Models									Goodness-of-fit	
	COEFFICIENTS (SE)								χ2	df
Linear-quadratic										
Y(D;C;α;β)	C = −0.0181	(0.0009)	α = 0.2480	(0.0081)	β = 0.0130	(0.0006)			746.37	6
Y(D;α;β)	__		α = 0.2431	(0.0080)	β = 0.0133	(0.0006)			742.19	7
Linear										
Y(D;C;α)	C = −0.0143	(0.0025)	α = 0.4125	(0.0059)	__				438.44	7
Y(D;α)	__		α = 0.4034	(0.0056)	__				875.31	8
GT										
Y(D; β_0_, β_1_, β_2_, β_3_)	β0 = 8.4716	(0.2097)	β1 = 6.8462	(0.1204)	β2 = 0.2318	(0.0051)	β3 = 1.0623	(0.1764)	70.14	6

### Dose estimates of simulated whole and partial body exposures

Table 4 shows the distribution of cells with dicentrics in the simulation of whole body (2, 6, 12 and 17 Gy) and partial body exposures (30% and 70% of irradiated blood at 6 and 12 Gy). As can be seen, underdispersion was observed for the whole body exposure simulations and a highly significant overdispersion, which increases as the percentage of irradiated blood decreases, was observed for partial body exposure simulations. In the simulation of whole body exposures, the estimated doses using the three mathematical models were in general close to the real dose (Table 5). With the linear and linear quadratic models, the doses were slightly overestimated, whereas with the GT model the doses were slightly underestimated. The dose estimates by the GT model were in three of the four doses more accurate than with the classical linear and linear quadratic ones. Moreover, in the simulation of partial exposures, the GT model also showed a better adjustment between the real dose and the estimated dose for the irradiated fraction.

**Table 4:** Cells analyzed and dicentrics distribution among cells for the simulated whole and partial body irradiations.

	% of		Dicentrics distribution among cells																				
Dose	irradiated blood	Cells	0	1	2	3	4	5	6	7	8	9	10	11	12	13	14	15	Dic	Y	SE	DI	U
2 Gy	100%	498	358	125	15	0	0	0	0	0	0	0	0	0	0	0	0	0	155	0.311	0.024	0.88	−1.83
	30%	400	369	2	6	7	7	8	1	0	0	0	0	0	0	0	0	0	109	0.273	0.051	3.75	38.97
6 Gy	70%	300	201	8	34	28	19	7	1	0	1	0	1	0	0	0	0	0	295	0.983	0.094	2.67	20.45
	100%	150	1	16	54	42	21	9	4	2	1	0	0	0	0	0	0	0	425	2.833	0.11	0.65	−3.06
	30%	250	233	0	0	1	2	2	8	1	1	1	1	0	0	0	0	0	103	0.412	0.101	6.15	57.71
12 Gy	70%	150	124	0	0	4	4	3	5	3	3	2	2	0	0	0	0	0	156	1.04	0.2	5.77	41.28
	100%	150	0	1	3	14	22	30	31	22	14	4	6	2	1	0	0	0	869	5.793	0.165	0.7	−2.57
17 Gy	100%	100	0	0	0	2	2	4	6	10	16	11	21	13	7	4	2	2	914	9.14	0.249	0.68	−2.25

Gy = Gray; Total dic = total number of dicentrics; Y = frequency of dicentrics; Var = variance; SE = standard error; DI = dispersion index (variance/mean); U = values of the u-test.

**Table 5:** Dose estimates and confidence intervals obtained by the three mathematical models.

	% of irradiated	Dose estimation in Gy (confidence interval)		
		Model		
Dose	blood	Linear (L)	Linear-quadratic (LQ)	Gompertz-type (GT)
2 Gy	100%	0.77 (0.64–0.93)	1.2 (0.96–1.51)	1.91 (1.67–2.16)
6 Gy	30%	8.43 (6.70–10.08)	9.28 (7.79–10.59)	6.57 (5.67–7.54)
	70%	6.94 (6.05–7.80)	8.01 (7.20–8.75)	5.86 (5.29–6.48)
	100%	7.02 (6.18–8.00)	8.08 (7.10–9.21)	5.66 (5.15–6.23)
12 Gy	30%	14.98 (12.05–17.89)	14.1 (12.05–15.88)	9.91 (8.20–11.99)
	70%	14.84 (12.48–17.17)	14 (12.35–15.45)	9.83 (8.37–11.54)
	100%	14.36 (13.00–15.89)	13.64 (12.37–15.10)	9.54 (8.72–10.45)
17 Gy	100%	22.66 (20.54–25.03)	18.62 (16.97–20.53)	16.37 (13.87–26.48)

## Discussion

### Standardization of the new method

The dicentric chromosome analysis has been used since the mid-1960s to estimate the dose of an exposure to ionizing radiation. The mathematical models developed have allowed the dose to be estimated taking into account different scenarios such as the type of ionizing radiation, the length of the exposure, the uniformity or not of the exposure and the time since the exposure [1]. However, the dicentric analysis usefulness has limitations for doses over 5 Gy because it is difficult to obtain enough metaphases to carry out confident dose estimation. For this reason the majority of dose-effect curves have been elaborated with doses up to 5 Gy. To avoid this limitation, some approaches have been suggested. Methods to analyze interphase cells by premature chromosome condensation [19], [20], [21], [22], [23] allows the chromosome analysis of heavily damaged cells that would remain blocked in the check-point G2/M. However, by this methodology the morphology of the chromosomes does not allow the observation of dicentrics by uniform stain, and for this reason the score of ring chromosomes and acentric chromosomes is proposed [20], [24], [25], [26]. Ring chromosomes are induced at a much lower frequency than dicentrics, making dose estimates less accurate. The increase of culture time is another approach to increase the number of analyzable cells. Different culture lengths have been used in different studies, 52 h [6], [11], [10], 56 h [27], 68 h [11],72 h [6], [27] and 96 h [6]. The frequencies of dicentrics and rings at different culture times were compared by some authors [6], [10], [11], [27] detecting no significant differences. Another approach to increase the number of analyzable cells at high doses is the use of caffeine to abrogate the G2/M checkpoint [12]. In the present study, performed with caffeine treated cultures, the frequency of dicentrics showed a progressive decrease from 54 h to 72 h (Figure 1). This was also observed in a previous study with caffeine where after a 15 Gy irradiation the frequency of dicentrics observed at 60 h of culture was significantly lower than one observed at 48 h [12]. It has been described that cells bearing dicentric chromosomes are preferentially eliminated in interphase by apoptosis [28]. For this reason, in caffeine treated cultures it is very important to obtain enough analyzable cells without compromising the frequency of dicentrics.

To perform the culture standardization, the dose of 10 Gy was selected because it is in the medium dose range that spans the projected dose-effect curve. It is known that irradiated cells show a delay in the cell cycle progression of approximately one hour per Gy of radiation received [29]. Therefore, in cultures irradiated at 10 Gy it is expected that the first wave of metaphases would be found at around 58 h of culture. We obtained the best relationship between MI and dicentric frequency with the 57 h cultures, and for this reason this culture time was chosen to elaborate the dose-effect curve.

### Underdispersion in the spotlight

A factor influencing the dose-effect curve fitting is the distribution of dicentrics among cells. In classical dose-effect curves for low LET radiation, this distribution fitted to a Poisson. However, at high doses of IR underdispersion in the dicentric distribution has been described by several authors [6], [10], [12], [30]. In the present study the underdispersion was observed at all doses, but with statistical significance at 3, 5, 7 and 10 Gy. To analyze this phenomenon, the observed cell distribution of dicentrics and the expected one assuming a Poisson distribution were calculated (Table 2), showing a clear tendency to detect fewer cells without or with fewer dicentrics than expected at doses higher than 5 Gy, where the number of cells without aberrations was lower than expected. A possible explanation could be related to the repair of double strand breaks (DSBs). It has been described that the probability for correct or incorrect DSBs rejoining depends on the spatial and temporal proximity of other DSBs [31], [32], [33], and consequently the increased probability of misrejoining at high doses could explain that the observed number of cells without or with few dicentrics was lower than expected for all doses from 5 to 25 Gy. On the other hand, at the highest doses, from 10 to 25 Gy, we observed fewer cells with elevated numbers of dicentrics than expected, probably due to a saturation of the dicentrics yield at very high doses [5], [6], [9], [10], [34]. This saturation in the formation of aberrations with centromere at very high doses is intrinsically limited by the number of centromeres available, which in a human cell is 46 [9], [35]. Both aspects the fewer cells than expected with low numbers of dicentrics and the saturation effect contributes to the underdispersion observed. It must be pointed out that a higher underdispersion at the medium doses (5–15 Gy) has also been observed by other authors [6], [9]. The tendency of the dicentrics frequency to saturate at the highest doses leads to a poor fitting with the linear and linear-quadratic models (Figure 3), where differences between the observed and expected values were observed in the entire dose range (Table 6). A similar poor fitting with the linear quadratic model had been previously described when high doses are analyzed [6], [10], [11].

**Table 6:** χ^2^ values for each dose for the number of dicentrics observed and expected for the three fitted models.

Dose (Gy)	Linear-quadratic	Linear	Gompertz-type
0	–	–	16.1
0.1	29.4	60.2	6.5
0.5	118.1	262.6	6.2
1	72.6	199.2	22.8
3	2.3	74.5	10.5
5	96.6	20.8	2.3
7	178.4	76.7	1.2
10	100.3	63.9	0
15	43.6	86.4	1.7
20	3.7	27.8	0.6
25	97.2	3.1	2.1
Total	742.2	875.3	70.1

### Establishment of a new dose-effect relationship

Taking into account the saturation of the dicentric frequencies for doses higher than 15 Gy, an empirical adjustment to a GT model was performed (Figure 3), and a better adjustment was obtained. For this model, the lower doses, up to 3 Gy were those accounting for the higher values in the chi-square statistic (Table 6).

Sasaki [9] proposed a semi-empirical multiparametric model based on a mixed Poisson distribution. However, from a mathematical point of view mixed Poisson distributions always are overdispersed [36] and consequently they do not allow to describe underdispersed scenarios. Parameters of Sasaki’s model were calculated in two steps: A first approximation to the classic linear-quadratic model to calculate the C, α and β coefficients using values obtained in the dose range 0.01–3 Gy; and then, keeping the obtained coefficients and using the whole dose range (up to 50 Gy) estimating 4 more parameters. One of them, a saturation constant parameter, was manually determined. In that multiparametric approach a slight deviation from the linear quadratic model was observed at low doses, leading to a possible underestimation in cases of dose assessment. In practice for dose assessment the author proposed the use of the classical linear-quadratic model for doses below 3 Gy, and the new multiparametric model for higher doses. It is worth mentioning that the model proposed here assumes a lesser number of coefficients (4), which are calculated in a single step. And although some deviations are observed in the low dose range, these are not always in the same sense. For example after 0.1 and 3 Gy the number of dicentrics observed was lower than expected (11 and 393 observed *vs* 23 and 463 expected respectively), but at 0.5 and 1 Gy the number of dicentrics observed was higher than expected (78 and 120 observed *vs* 59 and 78 expected respectively). As indicated below the obtained GT model accurately estimates the simulated 2 Gy irradiation.

The present GT model efficiency for dose estimations was tested in cases of simulated exposures. For whole body simulations, the dose estimate was clearly better with the GT model for three (2, 6, and 17 Gy) of the four doses when compared with the linear and linear-quadratic models. At 12 Gy a better fit was achieved with the linear-quadratic model but no model includes the real dose in its confidence interval. However, biological dosimetry in cases of suspected very high doses should be mainly used to dose estimation after partial or non-uniform exposures, because the majority of accidents involve non-uniform exposures [37], [38], [39], where the analyzed lymphocytes population contains a mixture of exposed and non-exposed cells. In conventional colcemid treated cultures it is widely accepted that dicentrics follow a Poisson distribution [30] and the mixture with non-irradiated cells causes overdispersion. In the present study, with caffeine and colcemid treatment, the dicentric distribution is underdispersed and overdispersion cannot be the single observation indicative of a partial body irradiation. Probably, irradiation to medium doses and to high fractions of the body will conform to Poisson distribution [3]. For this reason, the decision to apply the truncated weighted Poisson method should be taken not only considering the U-test, but also with information about the accident circumstances or from clinical symptoms that could appear, such as localized erythema. In the present study, when irradiated blood at 6 and 12 Gy was mixed with non-irradiated blood to simulate partial body irradiation of 30% and 70%, a clear overdispersion was observed, and the estimated doses in the partial body simulations were very close to the real dose.

In conclusion, the present study reveals that when caffeine is added to the cultures enough metaphases can be obtained to perform cytogenetic analyses for doses up to 25 Gy. With this treatment underdispersion is observed for all dicentric cell distributions. The calibration curve was adjusted to a GT weighted Poisson model to achieve a better fit in relation to the tendency to a saturation of dicentric frequency at high doses and the observed underdispersion. The results obtained for dose estimations of simulated whole and partial body exposures reaffirm the effectiveness of the achieved GT model and its usefulness for biological dosimetry.

## Supporting Information

File S1 **Figures S1–S4.** Figure S1. Outputs of the program “dose_response_curve.txt”. Figure S2. Plot of the dose-effect curve obtained using the R-program “dose_response_curve.txt”. Empty dots represent the observed frequencies of dicentrics and red line the obtained Gompertz function. Figure S3. Outputs of the program “wholebody_dose.txt”. The estimated dose is 5.66, and the 95% confidence interval is (5.15 −6.23). Figure S4. Outputs of the program “partialbody_dose.txt”. The estimated dose is 5.86, and the 95% confidence interval is (5.29 −6.48).
(PDF)
