# Correction: A New Model of Biodosimetry to Integrate Low and High Doses

**DOI:** 10.1371/journal.pone.0117767

**Published:** 2015-02-11

**Authors:** The PLOS ONE Staff

There are multiple errors in the equations in the last sentence of the third paragraph of the “Statistics” subsection of the Materials and Methods. The correct equations are:

$$Y_{0}-1.96\cdot SE\left(Y_{0}\right)=\hat{Y}\left(d_{L}\right)+R\cdot \sigma_{\hat{Y}}\left(d_{L}\right)$$

$$Y_{0}+1.96\cdot SE\left(Y_{0}\right)=\hat{Y}\left(d_{U}\right)-R\cdot \sigma_{\hat{Y}}\left(d_{U}\right)$$

Table 2 has been corrected for improved readability. Please see the corrected Table 2 here.

**Table 2:** Cells analyzed and dicentrics distribution among cells for the dose-effect curve. In brackets are shown the expected dicentrics distribution assuming a Poisson.

		**Dicentrics distribution among cells**																					
**Dose (Gy)**	**Cells**	**0**	**1**	**2**	**3**	**4**	**5**	**6**	**7**	**8**	**9**	**10**	**11**	**12**	**13**	**14**	**≥15#**	**Total Dic**	**Y**	**Var**	**SE**	**DI**	**U**
0	2000	1999	1	0	0	0	0	0	0	0	0	0	0	0	0	0	0	1	0.001	0.001	0.001	1.00	-
		(1998)	(2)	0	0	0	0	0	0	0	0	0	0	0	0	0	0						
0.1	2000	1989	11	0	0	0	0	0	0	0	0	0	0	0	0	0	0	11	0.006	0.005	0.002	0.99	−0.17
		(1988)	(12)	0	0	0	0	0	0	0	0	0	0	0	0	0	0						
0.5	2000	1922	78	0	0	0	0	0	0	0	0	0	0	0	0	0	0	78	0.039	0.037	0.004	0.96	−1.23
		(1924)	(75)	(1)	0	0	0	0	0	0	0	0	0	0	0	0	0						
1	1000	886	108	6	0	0	0	0	0	0	0	0	0	0	0	0	0	120	0.120	0.118	0.011	0.98	−0.43
		(887)	(106)	(6)	0	0	0	0	0	0	0	0	0	0	0	0	0						
3	500	213	192	85	9	1	0	0	0	0	0	0	0	0	0	0	0	393	0.786	0.641	0.036	0.82	−2.91
		(228)	(179)	(70)	(18)	(4)	(1)	0	0	0	0	0	0	0	0	0	0						
5	150	3	23	58	38	15	10	2	1	0	0	0	0	0	0	0	0	382	2.547	1.578	0.103	0.62	−3.29
		(12)	(30)	(38)	(32)	(21)	(10)	(4)	(2)	(1)	0	0	0	0	0	0	0						
7	150	0	4	23	35	35	29	10	9	4	1	0	0	0	0	0	0	604	4.027	2.697	0.134	0.67	−2.85
		(3)	(11)	(22)	(29)	(29)	(24)	(16)	(9)	(5)	(2)	(1)	0	0	0	0	0						
10	150	0	0	0	3	18	40	35	25	16	9	4	0	0	0	0	0	915	6.100	2.493	0.129	0.41	−5.11
		(1)	(4)	(11)	(19)	(25)	(26)	(23)	(17)	(12)	(7)	(4)	(2)	(1)	0	0	0						
15	100	0	0	0	0	3	10	12	21	10	16	7	7	7	3	1	3	834	8.340	6.792	0.261	0.81	−1.31
		0	0	(1)	(2)	(5)	(8)	(11)	(13)	(14)	(13)	(11)	(8)	(6)	(4)	(2)	(2)						
20	100	0	0	0	0	0	6	9	10	12	17	13	9	6	8	6	4	957	9.570	7.985	0.283	0.83	−1.17
		0	0	0	(1)	(2)	(5)	(7)	(10)	(12)	(13)	(12)	(11)	(9)	(6)	(4)	(6)						
25	100	0	0	0	0	0	4	5	5	8	18	16	12	7	3	9	13	1065	10.650	10.008	0.316	0.94	−0.42
		0	0	0	(1)	(3)	(5)	(7)	(10)	(12)	(12)	(12)	(11)	(9)	(7)	(5)	(8)						

Gy = Gray; Total dic = total number of dicentrics; Y = frequency of dicentrics; Var = variance; SE = standard error; DI = dispersion index (variance/mean); U = values of the u-test. # = Extended data: At 15 Gy, three cells with 15 dic were observed and one cell with 15 and 16 dic were expected; At 20 Gy, we observed one cell with 15 dic, one with 16 and two with 17. At this dose three cells with 15, two with 16 and one with 17 dic were expected. At 25 Gy we observed four cells with 15 dic, three with 16, three with 17, and three with 18. At this dose we expected three cells with 15 dic, two with 16 and one with 17, 18 and 19 respectively.

There is an error in Table 3. Please see the corrected Table 3 here.

**Table 3:** Dose-response coefficients obtained for the different adjustments to the models and their goodness-of-fit χ^2^ statistics.

**Models**									**Goodness-of-fit**	
	**COEFFICIENTS (SE)**								**χ^2^**	**df**
Linear-quadratic										
Y(D;C;α;β)	C = −0.0181	(0.0009)	α = 0.2480	(0.0081)	β = 0.0130	(0.0006)			746.37	6
Y(D;α;β)	—		α = 0.2431	(0.0080)	β = 0.0133	(0.0006)			742.19	7
Linear										
Y(D;C;α)	C = −0.0143	(0.0025)	α = 0.4125	(0.0059)	—				438.44	7
Y(D;α)	—		α = 0.4034	(0.0056)	—				875.31	8
GT										
Y(D; β_0_, β_1_, β_2_, β_3_)	β0 = 8.4716	(0.2097)	β1 = 6.8462	(0.1204)	β2 = 0.2318	(0.0051)	β3 = 1.0623	(0.1764)	70.14	6

## References

[1] Pujol M, Barquinero J-F, Puig P, Puig R, Caballín MR, Barrios L (2014) A New Model of Biodosimetry to Integrate Low and High Doses. PLoS ONE 9(12): e114137 doi:10.1371/journal.pone.0114137 25461738 10.1371/journal.pone.0114137 PMC4252095

