# Supplementary material for: A New Model of Biodosimetry to Integrate Low and High Doses
Source: PLoS One. 2014 Dec 2;9(12):e114137. doi: 10.1371/journal.pone.0114137 (PMC4252095; doi:10.1371/journal.pone.0114137)
Supplement: File S1 — Figures S1–S4. Figure S1. Outputs of the program “dose_response_curve.txt”. Figure S2. Plot of the dose-effect curve obtained using the R-program “dose_response_curve.txt”. Empty dots represent the observed frequencies of dicentrics and red line the obtained Gompertz function. Figure S3. Outputs of the program “wholebody_dose.txt”. The estimated dose is 5.66, and the 95% confidence interval is (5.15 −6.23). Figure S4. Outputs of the program “partialbody_dose.txt”. The estimated dose is 5.86, and the 95% confidence interval is (5.29 −6.48). (PDF) [file pone.0114137.s001.pdf]

Supplementary Material

This document contains the experimental codes of the 3 R-programs that have been used in the paper. The programs run well for the data shown in the paper. However, the codes are not still optimized, and some bugs could appear sometime analyzing other data sets.

1-dose_response_curve.txt

It estimates the parameters of the dose-effect curve using a Gompertz-type function and the weighted Poisson distribution allowing to model underdispersion.

The inputs are the observed dicentrics distributions among cells.

The main outputs are the estimates of the parameters, their standard errors and the variance-covariance matrix (Figure 1). The program also includes a plot of the dose-effect curve (Figure 2). The plot could be customized changing (or adding) titles, colours, etc. Run the command help (plot) to obtain information about the usage and the options of this graphical procedure.

2- wholebody_dose.txt

It calculates the estimated dose and a 95% confidence interval, assuming a whole body irradiation, using a Gompertz-type dose response curve.

The inputs are the parameters of the Gompertz-type dose response curve, the variance-covariance matrix and the observed dicentrics distribution.

The outputs are the estimated dose and the 95% confidence interval (Figure 3).

3- partialbody_dose.txt

It calculates the estimated dose and a 95% confidence interval, assuming a partial body irradiation, using a Gompertz-type dose response curve.

The inputs are the parameters of the Gompertz-type dose response curve, the variance-covariance matrix and the observed dicentrics distribution.

The main outputs are the estimated dose and the 95% confidence interval (Figure 4).

```
# dose_response_curve.txt

# Estimation of the dose-effect curve using a Gompertz type function

# and the weighted Poisson distribution shown in the paper in Equation 1.

#


# Reading data of the dicentrics distributions among cells for the dose-effect curve

# shown in the paper in Table 2

#

dose<-c(0, 0.1, 0.5, 1, 3, 5, 7, 10, 15, 20, 25)

ncel<-c(2000, 2000, 2000, 1000, 500, 150, 150, 150, 100, 100, 100)

nt<-length(dose)


 # Maximum number of dicentrics observed in a cell

maxd=18

dimens=nt*(maxd+1)

f=numeric(dimens)

dim(f) <- c(nt,maxd+1)

 #

# Columns of the dicentrics distribution

#

f[,1]= c(1999, 1989, 1922, 886, 213, 2, 0, 0, 0, 0, 0)

f[,2]= c(1, 11, 78, 108, 192, 23, 4, 0, 0, 0, 0)

f[,3]= c(0, 0, 0, 6, 85, 58, 23, 0, 0, 0, 0)

f[,4]= c(0, 0, 0, 0, 9, 38, 35, 3, 0, 0, 0)

f[,5]= c(0, 0, 0, 0, 1, 15, 35, 18, 3, 0, 0)

f[,6]= c(0, 0, 0, 0, 0, 10, 29, 40, 10, 6, 4)

f[,7]= c(0, 0, 0, 0, 0, 2, 10, 35, 12, 9, 5)

f[,8]= c(0, 0, 0, 0, 0, 1, 9, 25, 21, 10, 5)

f[,9]= c(0, 0, 0, 0, 0, 0, 4, 16, 10, 12, 8)

f[,10]=c(0, 0, 0, 0, 0, 0, 1, 9, 16, 17, 18)

f[,11]=c(0, 0, 0, 0, 0, 0, 0, 4, 7, 13, 16)

f[,12]=c(0, 0, 0, 0, 0, 0, 0, 0, 7, 9, 12)
```

```
f[,13]=c(0, 0, 0, 0, 0, 0, 0, 0, 7, 6, 7)
f[,14]=c(0, 0, 0, 0, 0, 0, 0, 0, 3, 8, 3)
f[,15]=c(0, 0, 0, 0, 0, 0, 0, 0, 1, 6, 9)
f[,16]=c(0, 0, 0, 0, 0, 0, 0, 0, 3, 1, 4)
f[,17]=c(0, 0, 0, 0, 0, 0, 0, 0, 0, 1, 3)
f[,18]=c(0, 0, 0, 0, 0, 0, 0, 0, 0, 2, 3)
f[,19]=c(0, 0, 0, 0, 0, 0, 0, 0, 0, 0, 3)

# Weigthed Poisson probability function
wpoiss=function(x,mu,b){(1+b*(x^2))*dpois(x,mu)/(1+b*(mu+mu^2))}

# Population mean of the Weigthed Poisson
meanwpoiss=function(mu,b){mu*(1+(b*(2*mu+1))/(1+b*(mu+mu^2)))}
#
# This is minus the log-likelihood function.
#
b=numeric(4)
loglik<-function(b){
loglik<-0
for(j in 1:nt){
lam<-b[1]*exp(-b[2]*exp(-b[3]*dose[j]))
for(i in 1:19){
loglik<-loglik+log(wpoiss(i-1,lam,b[4]*dose[j]))*f[j,i]
}}
-loglik}
#
# Procedure nlm finds the minimum (the MLE estimator).
# The initial values, in this case p=c(8.5,7.0,.3,0), have to be
# closed to the solution. Otherwise the numerical method
# could not converge. Several trials would be needed.
#
MLE<-nlm(loglik,p=c(8.5,7.0,.3,0),hessian=TRUE)
# Estimated parameters and its standard errors
```

```
MLE$estimate
sqrt(diag(solve(MLE$hessian)))
# Values of the gradient vector and convergence code
MLE$gradient
MLE$code
# Estimated variance-covariance matrix
solve(MLE$hessian)
#
# Plot of the observed frequency of dicentrics and the fitted Gompertz-type curve
#
mdic=numeric(nt)
for(i in 1:nt){mdic[i]=sum(f[i,]*(seq(1:19)-1))/ncel[i]}
lampred<-MLE$estimate[1]*exp(-MLE$estimate[2]*exp(-MLE$estimate[3]*dose))
pred=numeric(nt)
for(i in 1:nt){
pred[i]=meanwpoiss(lampred[i],MLE$estimate[4]*dose[i])}
plot(dose,mdic)
lines(dose,pred,col="red")
```

```
R Console
> # Estimated parameters and its standard errors
>   MLE$estimate
[1] 8.4715598 6.8461972 0.2317881 1.0622618
>   sqrt(diag(solve(MLE$hessian)))
[1] 0.209652185 0.120360791 0.005056807 0.176418972
>   # Values of the gradient vector and convergence code
>   MLE$gradient
[1]  4.031315e-05 -9.870510e-05  1.227818e-03  1.258595e-04
>   MLE$code
[1] 1
>   # Estimated variance-covariance matrix
>   solve(MLE$hessian)
              [,1]         [,2]          [,3]          [,4]
[1,]  0.0439540386 0.0027089464 -6.928684e-04  5.825566e-03
[2,]  0.0027089464 0.0144867200  2.018754e-04  1.485129e-02
[3,] -0.0006928684 0.0002018754  2.557129e-05 -6.760215e-05
[4,]  0.0058255661 0.0148512940 -6.760215e-05  3.112365e-02
> |
```

Figure S1. Outputs of the program "dose_response_curve.txt".

- The estimates of the parameters of the dose-effect curve and their standard errors are,

β0= 8.47156±0.20965

β1= 6.84620±0.12036

β2= 0.23179±0.00506

β3= 1.06226±0.17642

- The values of the gradient vector are closed to zero:

4.031e-05 -9.870e-05 1.228e-03 1.258e-04

This is indicative that the estimates of the parameters maximize the log-likelihood function.

If the gradient vector were not closed to zero the solution could not be valid. Then, it is suggested to run again the program with different initial values in the nlm procedure.

- MLE$code gives an integer indicating why the optimization process of the nlm procedure has finished. A value of 1 or 2 indicates that the recursive numerical procedure finally provides a reliable solution. For other values the solution could not be valid and it is suggested to run again the program with different initial values.

- The estimated variance-covariance matrix will be needed to calculate confidence limits.

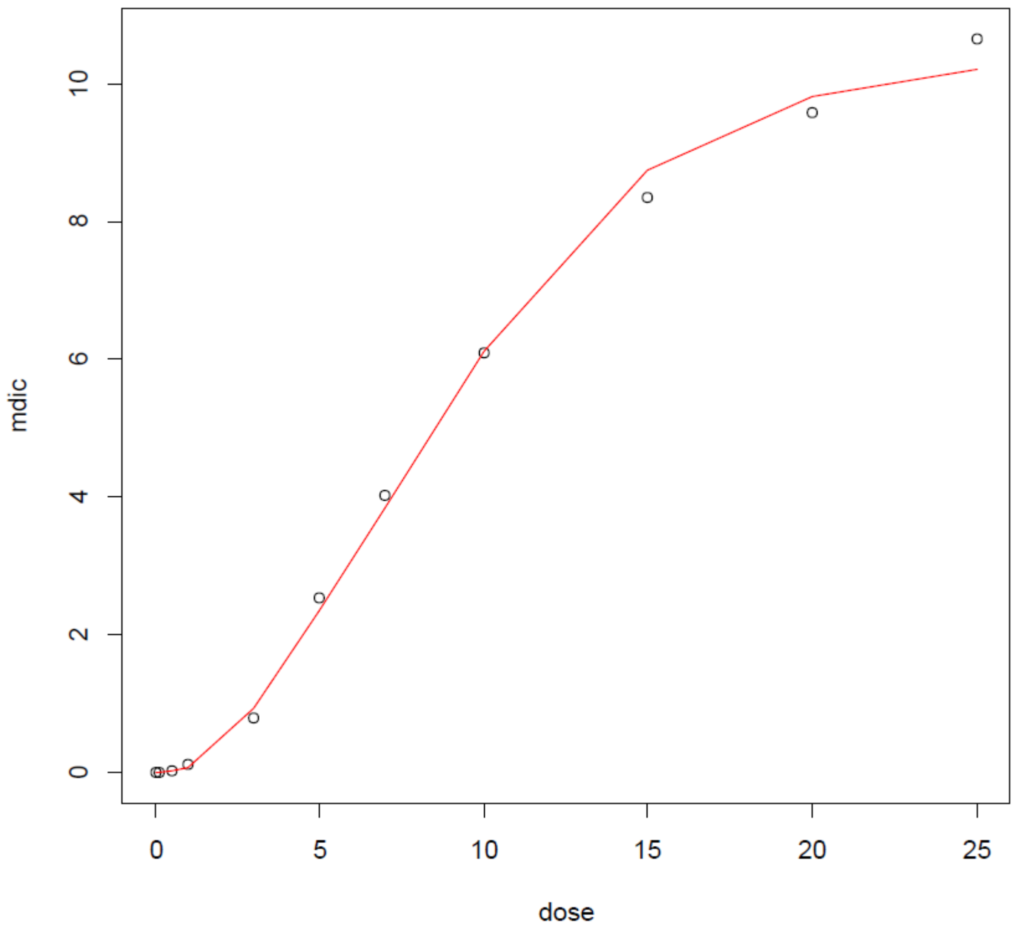

Figure S2. Plot of the dose-effect curve obtained using the R-program “dose_response_curve.txt”. Empty dots represent the observed frequencies of dicentrics and red line the obtained Gompertz function.

The plot can be customized changing (or adding) titles, colours, etc. Run the command help (plot) to obtain information about the usage and the options of this graphical procedure.

```
# wholebody_dose.txt

# This program calculates the estimated dose and a confidence interval

# A whole body irradiation is assumed

#

# Input of the parameters of the dose-effect Gompertz-type model

beta0=8.47156; beta1=6.84620; beta2=0.23179; beta3=1.06226


# Input of the Variance-covariance matrix of the parameters

sigma=numeric(16)

dim(sigma) <- c(4,4)

sigma[1,1]=0.043954; sigma[2,2]=0.014487; sigma[3,3]=0.000026; sigma[4,4]=0.031124

sigma[1,2]=0.002709; sigma[1,3]=-0.000693; sigma[1,4]=0.005826

sigma[2,1]=sigma[1,2]; sigma[2,3]=0.000202; sigma[2,4]=0.014851

sigma[3,1]=sigma[1,3];sigma[3,2]=sigma[2,3];sigma[3,4]=-0.0000676

sigma[4,1]=sigma[1,4];sigma[4,2]=sigma[2,4];sigma[4,3]=sigma[3,4]


# Population mean of the Weigthed Poisson and prediction function.

meanwpoiss=function(mu,b){mu*(1+(b*(2*mu+1))/(1+b*(mu+mu^2)))}

pr=function(d){meanwpoiss(beta0*exp(-beta1*exp(-beta2*d)),beta3*d)}


# This function calculates the variance of the predictions using the

# delta-method

varpr=function(d){

predder <- deriv(~b1*exp(-b2*exp(-b3*x))*(1+(b*x*(2*b1*exp(-b2*exp(-
b3*x))+1))/(1+b*x*(b1*exp(-b2*exp(-b3*x))+(b1*exp(-b2*exp(-
b3*x)))^2))),c("b1","b2","b3","b"),function(x,b1,b2,b3,b) NULL)

grad<-attr(predder(d,beta0,beta1,beta2,beta3),"gradient")

grad%*%sigma%*%t(grad)}


# Reading data of the dicentrics distribution which the dose has to be estimated

# The example corresponds to the 6G simulated whole body irradiation

# shown in Table 4.

# Maximum number of dicentrics observed in a cell
```

```
max=8

nn=max+1

g=numeric(nn)

g[1]=1;g[2]=16;g[3]=54;g[4]=42;g[5]=21;g[6]=9;g[7]=4;g[8]=2;g[9]=1


# Mean and standard deviation calculations

me=sum(g[1:nn]*seq(0,(nn-1))[1:nn])/sum(g[1:nn])

va=(sum(g[1:nn]*seq(0,(nn-1))[1:nn]^2)/sum(g[1:nn])-
me*me)*sum(g[1:nn])/(sum(g[1:nn])-1)

s=(va/sum(g[1:nn]))^.5


# Procedure uniroot solves the equations of the adapted Merkle’ s approach described in the
# paper. The interval (0.1,30) has been chosen because there is a change of sign of the

# objective functions in the extremes of this interval. Could be necessary to change this

#  interval for other data sets.

ds=uniroot(function(d){pr(d)-me},c(0.1,30))$root

ds

ll=uniroot(function(d){pr(d)+sqrt(qchisq(0.95,4)*varpr(d))-me+1.96*s},c(0.1,30))$root

ul=uniroot(function(d){pr(d)-sqrt(qchisq(0.95,4)*varpr(d))-me-1.96*s},c(0.1,30))$root

c(ll,ul)
```

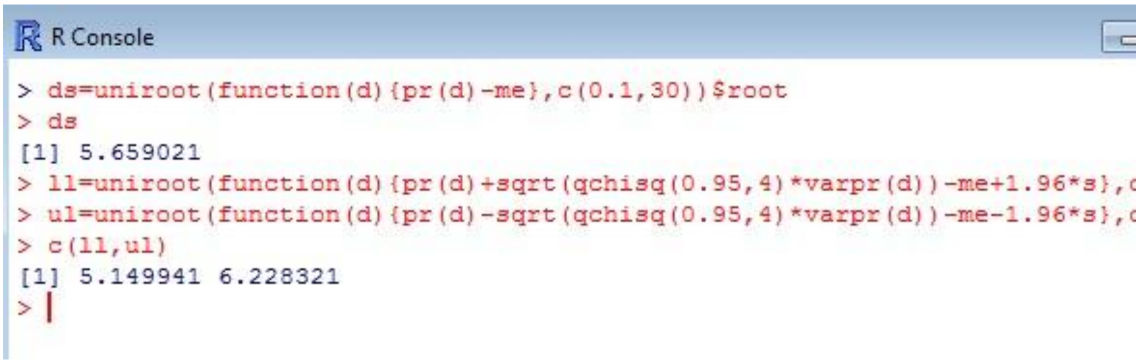

```
R Console
> ds=uniroot(function(d){pr(d)-me},c(0.1,30))$root
> ds
[1] 5.659021
> ll=uniroot(function(d){pr(d)+sqrt(qchisq(0.95,4)*varpr(d))-me+1.96*s},c
> ul=uniroot(function(d){pr(d)-sqrt(qchisq(0.95,4)*varpr(d))-me-1.96*s},c
> c(ll,ul)
[1] 5.149941 6.228321
>
```

Figure S3. Outputs of the program “wholebody_dose.txt”. The estimated dose is 5.66, and the 95% confidence interval is (5.15 - 6.23).

```
# partialbody_dose.txt

# This program calculates the estimated doses and a confidence interval

# A partial body irradiation is assumed

#

# Input of the parameters of the dose-effect Gompertz-type model

beta0=8.47156; beta1=6.84620; beta2=0.23179; beta3=1.06226


# Input of the Variance-covariance matrix of the parameters

sigma=numeric(16)

dim(sigma) <- c(4,4)

sigma[1,1]=0.043954; sigma[2,2]=0.014487; sigma[3,3]=0.000026; sigma[4,4]=0.031124

sigma[1,2]=0.002709; sigma[1,3]=-0.000693; sigma[1,4]=0.005826

sigma[2,1]=sigma[1,2]; sigma[2,3]=0.000202; sigma[2,4]=0.014851

sigma[3,1]=sigma[1,3];sigma[3,2]=sigma[2,3];sigma[3,4]=-0.0000676

sigma[4,1]=sigma[1,4];sigma[4,2]=sigma[2,4];sigma[4,3]=sigma[3,4]


# Weigthed Poisson probability function

wpoiss=function(x,mu,b){(1+b*(x^2))*dpois(x,mu)/(1+b*(mu+mu^2))}


# Population mean of the Weigthed Poisson and prediction function.

meanwpoiss=function(mu,b){mu*(1+(b*(2*mu+1))/(1+b*(mu+mu^2)))}

pr=function(d){meanwpoiss(beta0*exp(-beta1*exp(-beta2*d)),beta3*d)}


# This function calculates the variance of the predictions using the

# delta-method

varpr=function(d){

predder <- deriv(~b1*exp(-b2*exp(-b3*x))*(1+(b*x*(2*b1*exp(-b2*exp(-
b3*x))+1))/(1+b*x*(b1*exp(-b2*exp(-b3*x))+(b1*exp(-b2*exp(-
b3*x)))^2))),c("b1","b2","b3","b"),function(x,b1,b2,b3,b) NULL)

grad<-attr(predder(d,beta0,beta1,beta2,beta3),"gradient")

grad%*%sigma%*%t(grad)}


# Reading data of the dicentrics distribution which the dose has to be estimated
```

```
# The example corresponds to the 6G simulated partial body irradiation with 70%

# of irradiated blood, shown in Table 4.

# Maximum number of dicentrics observed in a cell

max=10

nn=max+1

g=numeric(nn)

g[1]=201;g[2]=8;g[3]=34;g[4]=28;g[5]=19;g[6]=7;g[7]=1;g[8]=0;g[9]=1;

g[10]=0;g[11]=1


# This is minus the log-likelihood function of the truncated at zero weighted Poisson.

loglik<-function(b){

loglik<-0

for(i in 2:nn){

loglik<-loglik+log(wpoiss(i-1,b[1],b[2])/(1-wpoiss(0,b[1],b[2])))*g[i]}

-loglik}

#

# Procedure nlm finds the minimum (the MLE estimator).

# The initial values, in this case p=c(1.4,2000), have to be

# closed to the solution. Otherwise the numerical method

# could not converge. Several trials would be needed.

#

MLE<-nlm(loglik,p=c(1.4,20000),hessian=TRUE)

# Mean and standard deviation estimations

me=meanwpoiss(MLE$estimate[1],MLE$estimate[2])

meander <- deriv(~mu*(1+(b*(2*mu+1))/(1+b*(mu+mu^2))),c("mu","b"),function(mu,b)
NULL)

grad3<-attr(meander(MLE$estimate[1],MLE$estimate[2]),"gradient")

s=(grad3%*%solve(MLE$hessian)[1:2,1:2]%*%t(grad3))^.5


# Procedure uniroot solves the equations of the adapted Merkle’ s approach described in the
# paper. The interval (0.1,30) has been chosen because there is a change of sign of the

# objective functions in the extremes of this interval. Could be necessary to change this

#  interval for other data sets.

ds=uniroot(function(d){pr(d)-me},c(0.1,30))$root
```

```
ll=uniroot(function(d){pr(d)+sqrt(qchisq(0.95,4)*varpr(d))-me+1.96*s},c(0.1,30))$root

ul=uniroot(function(d){pr(d)-sqrt(qchisq(0.95,4)*varpr(d))-me-1.96*s},c(0.1,30))$root

ds

c(ll,ul)

# Values of the gradient vector and convergence code

MLE$gradient

MLE$code
```

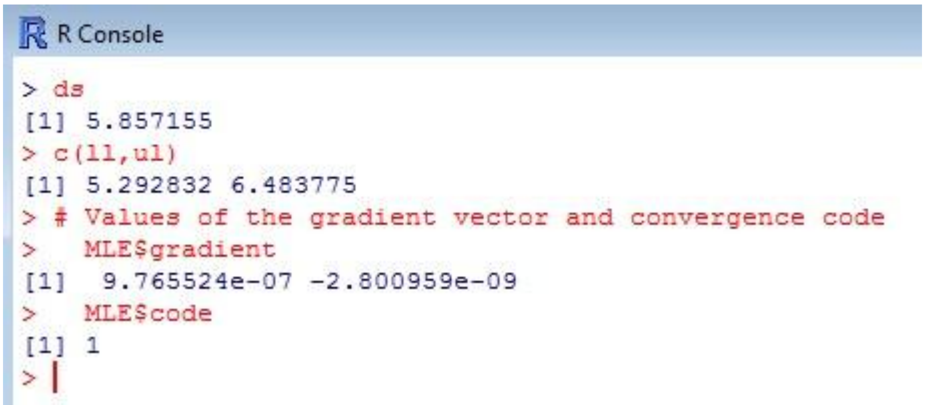

Figure S4. Outputs of the program "partialbody_dose.txt". The estimated dose is 5.86, and the 95% confidence interval is (5.29 - 6.48).

- The values of the gradient vector are closed to zero:

  9.765e-07 -9.801e-09

  This is indicative that the estimates of the parameters maximize the log-likelihood function.

  If the gradient vector were not closed to zero the solution could not be valid. Then, it is suggested to run again the program with different initial values in the nlm procedure.

- MLE$code gives an integer indicating why the optimization process of the nlm procedure has finished. A value of 1 or 2 indicates that the recursive numerical procedure finally provides a reliable solution. For other values the solution could not be valid and it is suggested to run again the program with different initial values.
